# Surgical procedures and outcomes in patients with tear drainage system disorders associated with the anticancer agent S-1

**DOI:** 10.1007/s10384-026-01377-6

**Published:** 2026-06-11

**Authors:** Tsugihisa Sasaki, Tsutomu Sounou, Tomomi Higashide

**Affiliations:** 1 Sasaki Eye Clinic, 5-2-6 Mikuni-higashi, Mikuni, Sakai, 913-0016 Japan; 2 Department of Ophthalmology, Keijyu Kanazawa Hospital, 6-26 Shimoshinmachi, Kanazawa, Japan; 3 Department of Ophthalmology, Kanazawa University School of Medicine, 13-5 Takara-machi, Kanazawa, Japan

**Keywords:** S-1, Canalicular obstruction, Dacryoendoscope, Canalicular sclerosis, Exploratory horizontal part canaliculotomy

## Abstract

**Purpose:**

S-1 is a first-line oral anti-cancer drug widely used in Japan. This study evaluated surgical outcomes for S-1–associated tear drainage system disorders (S-1/TDSD) and explored strategies to improve outcomes.

**Study design:**

Multicenter, retrospective observational study.

**Methods:**

Fifty-one patients (89 sides; 31 men and 20 women; mean age ± SD, 68.7 ± 10.7 years; range 49─85 years) with S-1/TDSD were included. Surgical procedures performed were: three-snip procedure; exploratory horizontal part canaliculotomy; probing with tube placement; and conjunctivodacryocystorhinostomy with Jones tube insertion.

**Results:**

The cases were classified as: punctal occlusion (Group a, 34 sides), both stenosed canaliculi (Group b, 2 sides), patent canaliculus and mono-canalicular obstruction (CO) (Group c, 9 sides), one soft or tightCO and contralateral softCO (Group d, 13 sides), tightCOs in both canaliculi (Group e, 27 sides), and common CO (Group f, 4 sides). The three-snip procedure for Group a was successful in 32 of 34 sides (94.1%). Probing with intubation for Group b was successful in all cases (2/2). Conjunctivodacryocystorhinostomy for Group e was successful in 15 of 20 sides (75.0%) and dacryoendoscopic canaliculo-incision for Group e was successful in 4 of 4 sides (100%). However, exploratory horizontal part canaliculotomy and probing for Groups c and d were successful in 14%─42% of cases.

**Conclusion:**

The three-snip procedure for punctal occlusions showed favorable outcomes but procedures preserving the canaliculus, especially in patients with horizontal CO, did not yield good results, even for an affected single canaliculus.

**Supplementary Information:**

The online version contains supplementary material available at https://doi.org/10.1007/s10384-026-01377-6.

## Introduction

S-1 is an oral drug composed of tegafur (a prodrug of 5-FU), gimeracil (a 5-FU metabolism modulator), and oteracil. In Japan it is primarily recommended for patients with unresectable or recurrent gastric cancer and colorectal cancer [1]. Esmaeli et al. first reported on S-1-related tear drainage system disorders (S-1/TDSDs) in 2005, which occurred during a clinical trial involving a combination of S-1 and cisplatin. They observed lacrimal dysfunction in three patients [2]. A retrospective study involving 55 patients treated with S-1 for more than one month showed severe stenosis or obstruction at the punctum, canaliculus, or nasolacrimal duct in 6 of 55 (8~11%) patients [3, 4]. Kim reports that 53 of 145 (37%) patients receiving adjuvant S-1 therapy for gastric cancer developed epiphora, and 88% of those patients were diagnosed with obstruction of the nasolacrimal duct [5].

S-1/TDSDs present a few treatment challenges: first, patients with severe S-1/TDSDs need to undergo surgical intervention; [4─7] second, degrees of occlusion vary widely, and the appropriate surgical interventions for the types of occlusion remain unclear. With the aim of improving surgical outcomes, this retrospective study investigated the types and frequencies of canalicular stenosis (CS) and canalicular obstructions (COs) in patients with S-1/TDSDs and evaluated the surgical interventions for patients according to each type of occlusion.

## Methods

### Patients

A total of 51 patients (89 sides) diagnosed with S-1/TDSDs, including individuals with gastric, colon, pancreatic, or lung cancer (31 men and 20 women; ages 49-85 years; mean age ± SD, 68.7 ± 10.7 years), were enrolled. Exclusion criteria included a history of facial trauma, lid anomalies, prior lacrimal surgery; and prior use of anti-glaucoma eyedrops, docetaxel, paclitaxel, or intravenous 5-FU. All the study patients were evaluated and treated at Keijyu Kanazawa Hospital or Sasaki Eye Clinic between 2007 and September 2023. Medical records were used as an additional source of information. Each patient received S-1 orally (30 mg/m^2^ every 12 hours) for over 28 consecutive days. Informed consent was obtained from each participant, and the study was approved by the Institutional Review Board of Keijyu Kanazawa Hospital. The study adhered to the tenets of the Declaration of Helsinki.

### Patient evaluations

The patient evaluation methods were based on the methods described in our previous report on S-1/TDSD [4]. Patients were closely examined by an ophthalmologist (T.S.) for eye complaints or findings such as tearing, discharge, eye pain, or blurred vision complained of at a prior visit with their referring physicians. Patients underwent a comprehensive ophthalmologic examination that included tear meniscus height (TMH) measurement evaluation with a photo slit lamp microscope (700GL; Takagi Seiko Co.,), fluorescein staining of the ocular surface, lacrimal probing, lacrimal irrigation, and the Schirmer test.

The postoperative evaluation was primarily performed 1 year after surgery. However, cases judged as failures 3 months postoperatively were classified as failures without waiting until the 1-year follow-up evaluation. Postoperative evaluations were based on whether patency was achieved and whenever the TMH was ≤ 0.4 mm, that was considered a success. Patency alone was considered a moderate success.

During follow-up visits, patients were asked about epiphora. The patients who underwent conjunctivodacryocystorhinostomy (ConjDCR) with Jones tube insertion and developed elevated TMH or continuous epiphora, underwent additional examinations. Dacryoendoscopy was performed in patients with severe symptoms (i.e., epiphora or discharge), despite high lacrimal patency.

**Fig. 1:**
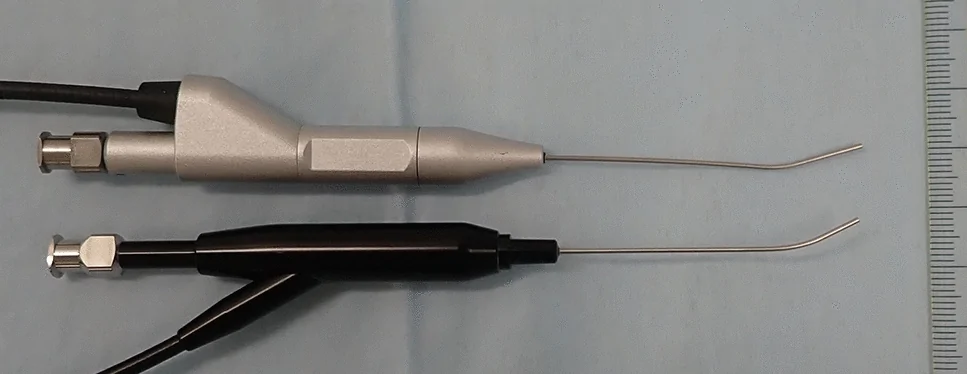
Lacrimal endoscopic handpieces (upper: LAC-06FY, Machida Endoscope Company Limited, lower: CK-10, Fibertech)

The dacryoendoscopic procedures used in this study have been described elsewhere [8]. Briefly, after instillation of oxybuprocaine and infra-trochlear anesthesia, the upper and lower lacrimal puncta were dilated and incised laterally, and the dacryoendoscope (CK-10, FT-203F; Fibertech Co.; or LAC-06FY, MVH-2010A equipped with the RLED-81 light source, Machida Endoscope Co.; Fig. 1) was inserted through the upper and lower canaliculi. Saline was injected rhythmically through the water channel for clear viewing of the lumen of the duct and evaluation of its extensibility. The positions and degrees of CS/CO were identified by dacryoendoscopy and simultaneous probing.

### CS and CO classifications

The classifications of CS and CO in S-1/TDSDs were based on the hardness of the obstruction and its location, evaluated by probing and irrigation with a 23G Bangerter lacrimal probe cannula. CS was defined as a caliber smaller than the outer diameter of the dacryoendoscope (0.9 mm, Fig.1) after inflation by hydropressure fluctuation from the water channel of the dacryoendoscope with a lacrimal irrigation patency [4]. If the horizontal canalicular obstruction could easily be opened and the distal lacrimal duct was patent, it was designated a soft canalicular obstruction (softCO). Obstructions that were opened with difficulty or multiple obstructions in a canaliculus were defined as tightCOs.

**Fig. 2:**
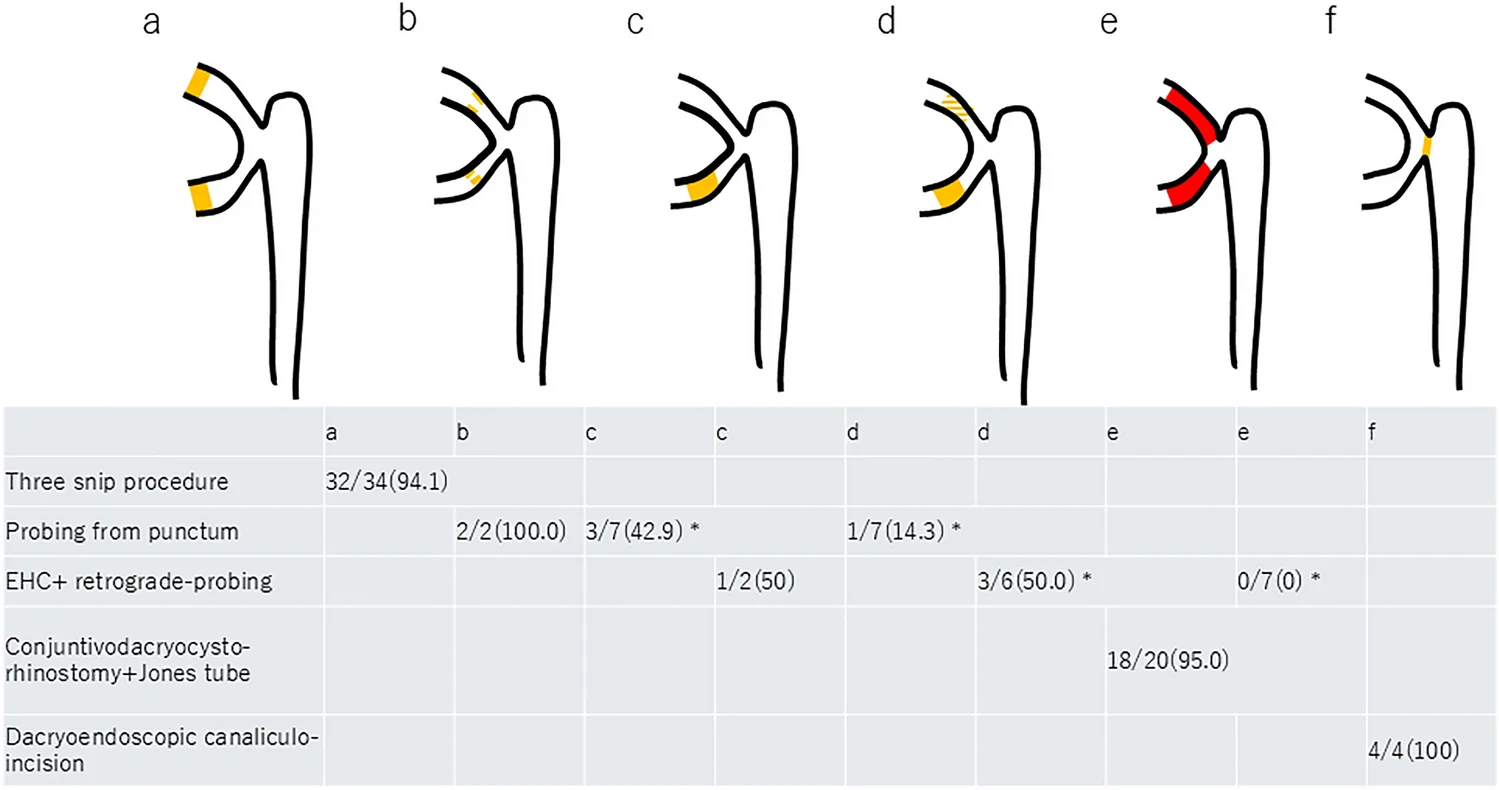
Schematic illustrations of six patterns of canalicular obstruction (CO) (Groups a–f), with the corresponding surgical procedures and outcomes. Group a represents punctal obstruction treated with the three-snip procedure. Group b indicates proximal canalicular stenosis treated with probing through the punctum. Group c represents horizontal CO treated with probing or exploratory horizontal part canaliculotomy (EHC) with retrograde or anterograde probing. Group d indicates soft CO with contralateral obstruction treated with probing or EHC combined with retrograde or anterograde probing. Group e represents complete CO requiring conjunctivodacryocystorhinostomy (ConjDCR) with Jones tube placement. Group f indicates common CO treated with dacryoendoscopic canaliculo-incision. Yellow areas indicate CO. Hatched yellow areas represent soft CO and red areas represent tight CO. Numbers indicate the number of successful cases over the total number of eyes with percentages shown in parentheses. An asterisk (*) indicates a statistically significant difference compared to Group a after a Holm correction (adjusted *P* < 0.05). Surgical success was defined as complete or moderate success and was analyzed as a binary outcome

Patients who had only obstruction/stenosis of the upper and lower lacrimal puncta were classified into Group a (Fig. 2a). Patients with bilateral stenosed canaliculi were classified into Group b (Fig. 2b). Patients with a patent or stenosed canaliculus and monocanalicular soft or tightCO were classified into Group c (Fig. 2c). Patients with monocanalicular soft or tightCO and contralateral softCO were classified into Group d (Fig. 2d). Patients with tightCOs in both canaliculi were classified into Group e (Fig. 2e). Patients with common canalicular obstructions were classified into Group f (Fig. 2e).

### Surgical interventions for CS and CO

**Fig. 3:**
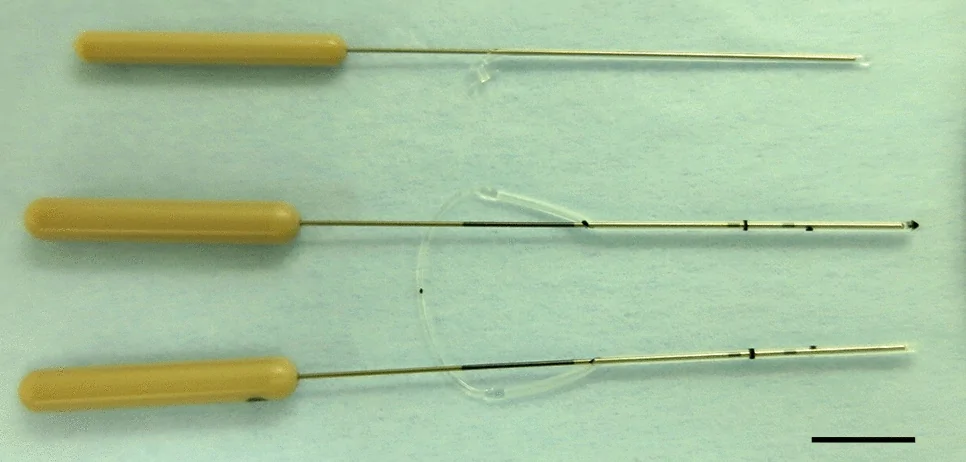
Two types of lacrimal tubes. Upper: Monocanalicular tube (Masterka; FCI Opthalmics, Paris, France); Lower: bicanalicular silicone tubes (Nunchaku-style silicone tubes; FCI Opthalmics). Scale:10 mm

All Group a patients underwent a three-snip procedure followed by insertions of bicanalicular lacrimal tubes (Nunchaku-style Silicone Tube [NST]; FCI Opthalmics; [Fig 3]). The three-snip procedure was then performed for patients with co-existing punctum closure/stenosis in Groups b─f. Group b patients underwent lacrimal probing for CS followed by intubation. The stenotic area was progressively dilated using a Bowman’s dilator for CS and NST or monocanalicular tube [monoT] (Masterka; FCI Opthalmics; [Fig. 3]) placement was performed.

Group c patients underwent lacrimal probing for CS/CO. Exploratory horizontal part canaliculotomy (EHC) + probing were performed if probing from the punctum was impossible because of CS/CO.

**Fig. 4:**
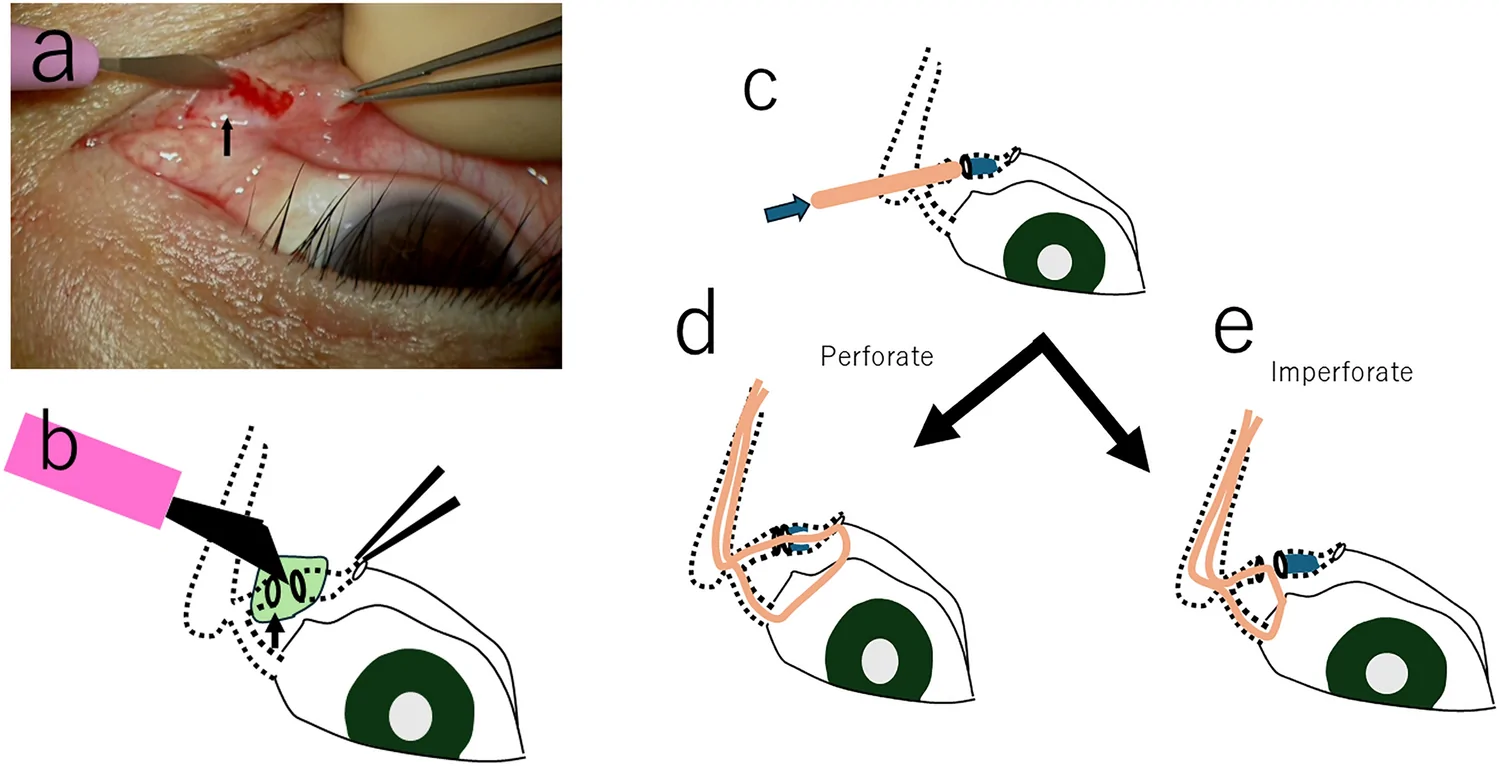
Surgeon’s view of exploratory horizontal part canaliculotomy (EHC) and a schematic image of EHC. **a**. Surgeon’s view of EHC (right side). A vertical incision is made on the long axis of the horizontal section of the canaliculus. Arrow: cross section of the lacrimal canaliculus. **b**. Schematic image of EHC. **c**. Schematic image of perforated retrograde probing from the cut end of a canaliculotomy. **d**. Schematic image of perforated retrograde probing after EHC followed by tubing from the lower punctum. **e**. Schematic image of imperforated retrograde probing after EHC followed by bypass tubing from a new punctum

EHC + probing was performed as follows: a vertical incision was made on the long axis of the horizontal section of the canaliculus at a point 4–5 mm medial to the punctum (Fig. 4. a, b). Retrograde probing was performed from the proximal end of the canaliculus to the punctum if the proximal canaliculus was closed. Either an NST or monoT was inserted through the punctum. Whenever the distal canaliculus was closed, anterograde probing was performed from the cut end of the distal canaliculus. An NST was inserted through the new punctum to bypass the proximal CO in case of an unopened proximal CO (Fig. 4).

EHC + probing, followed by placements of an NST or monoT into the upper and/or lower canaliculi, were performed for Group d patients. For Group e patients, for whom forceful probing could not achieve perforation, ConjDCR with Jones tube placement was performed. If patency could be restored by forceful probing or EHC + probing, the type of lacrimal tube that was used (monoT or NST) was based on the number of patent canaliculi. For group f patients with common canalicular obstructions, dacryoendoscopic canaliculo-incisions followed by NST placement were performed (Fig. 1) [9].

### Statistical analysis

Surgical success was defined as complete or moderate success and was analyzed as a binary outcome. Associations between group classification and surgical success were initially evaluated using an extended Fisher’s exact test because of the small sample sizes and sparse data in several groups. Because it (three-snip procedure for punctal obstruction) represented the standard procedure with the largest sample size, Group a was prespecified as the reference group for pairwise comparisons.

*Post hoc* pairwise comparisons between Group a and each of the other groups were performed using Fisher’s exact test. *P* values were adjusted using Holm’s method to control for a type I error due to multiple comparisons. A two-sided adjusted *P* value < 0.05 was considered statistically significant. All statistical analyses were performed using EZR (Saitama Medical Center, Jichi Medical University), a graphical user interface for R.

## Results

All cases involved disorders of the punctum or canalicular system, and no cases showed obstruction of the lacrimal sac or nasolacrimal duct. The types of obstruction in the study patients are shown in Fig. 2. The number of patients and sides in each group were: Group a, 34 sides and 18 patients; Group b, 2 sides and 1 patient; Group c, 9 sides and 6 patients; Group d, 13 sides and 8 patients; Group e, 27 sides and 16 patients; and Group f, 4 sides and 2 patients.

Group a demonstrated a high success rate with the three-snip procedure being successful in 32 of 34 sides (94.1%). All sides in Group b achieved successful outcomes following probing and NST intubation (2/2 [100%]). EHC + retrograde probing followed by NST intubation was successful in 1 of 2 sides (50%) in Group c, while probing from punctum followed by 3 pairs of NST and 4 monoT intubations resulted in success in 3 of 7 sides (42.9%).

Group d had poorer outcomes with anterograde probing from punctum and tube placement for soft CO with contralateral obstruction successful in only 1 of 7 sides (14.3%). EHC + retrograde probing followed by intubation in Group d achieved success in 2 of 6 sides (33.3%) with moderate success observed in an additional 1 side (16.7%). Only one canaliculus (either upper or lower) could be recanalized in three cases of EHC + retrograde probing requiring monoT tube insertion. Postoperative failure occurred in all three cases. Newly created punctum in successful cases of EHC + retrograde probing demonstrated a pinhole-like punctum.

Conjunctivodacryocystorhinostomy (ConjDCR) with Jones tube placement in Group e, was performed on 20 sides, yielding success in 15 sides (75.0%), including moderate success in 3 sides (20%). All unsuccessful cases in the ConjDCR with Jones tube placement group were attributable to burial or extrusion of the Jones tube.

All remaining sides in Group e treated with EHC + forceful retrograde probing followed by tube placement were classified as failures.

Group f had the most favorable outcomes with dacryoendoscopic canaliculo-incision combined with the three-snip procedure that proved successful in all sides (4/4 [100%]).

Using Group a as the reference, Fisher’s exact test with the Holm correction had significantly lower success rates in the probing group of Group c, in all cases of Group d, and in the probing group of Group e (adjusted *P* < 0.05; Fig. 2).

The mean duration of S-1 administration until the initial ophthalmologic consultation was 5.3 ± 3.7 months.

## Discussion

S-1 is widely used in Japan for malignancies such as gastric, lung, and colorectal cancer, and its irreversible side effects regarding visual function has had a marked impact on the treatment of cancer. To our knowledge, this study is the first to address the indications and 1-year surgical outcomes of patients with S-1/TDSD. Most of our study cases of S-1/TDSD involved damage to the lacrimal puncta and canaliculi. There were many variations of canalicular sclerosis and stenosis associated with S-1/TDSD. Surgical outcomes for each type of obstruction showed that puncture and tube placement for punctal closures alone (Group a) produced relatively high success rates 94.1% (32 of 34 cases). Jones tube placement also resolved tight obstructions of both the upper and lower horizontal canaliculi in approximately 90% (18 of 20 cases). The success of surgery for cases of horizontal canalicular obstruction in only one of the upper or lower ducts was less than 50%. The remaining cases required reoperation via ConjDCR.

These results suggest that surgical outcomes vary significantly according to the subtype of punctual or canalicular disorder, highlighting the importance of selecting procedure-specific strategies tailored to obstruction characteristics.

S-1 is an analogue of 5-FU, designed to reduce gastrointestinal side effects and increase the blood levels of 5-FU. Therefore, its side effects outside the gastrointestinal system are presumed to follow patterns similar to those caused by 5-FU. Patterns of lacrimal obstruction caused by 5-FU include not only punctal and canalicular obstructions but also sclerosis and stenosis of patent canaliculi [10–13], patterns similar to those of obstruction observed in this study.

The obstruction of the lacrimal drainage system associated with 5-FU treatment is often irreversible, even if the agent is discontinued [10]. The onset typically occurs 2 to 9 weeks after the initiation of the drug, occurring with frequencies of 3%─15% [11, 14], similar to the rates observed in patients with S-1. Furthermore, postoperative re-obstruction is common with 5-FU, and often the surgery requires ConjDCR, which is a feature shared with S-1/TDSD [13–15].

This study shows that patients with S-1/TDSD, even those with horizontal canalicular obstruction involving only one duct preoperatively (Group c), had insufficient function of the canaliculus as tearing or discharge preoperatively.

The results might be accounted for by the fact that S-1 not only causes obstruction but also could lead to sclerosis of the patent lacrimal duct, decreased function of the lacrimal drainage system [16], and inflammation around the canaliculus [17].

Previous reports on lacrimal drainage obstruction associated with systemic 5-fluorouracil and S-1 therapy suggest that inflammatory and cicatricial changes may persist or progress even after discontinuation of the causative agent [18, 19].

Tear drainage disorders associated with S-1 and other anti-cancer agents show a wide spectrum of canalicular involvement. In clinical practice the pattern of obstruction is not uniform and may involve different segments of the canaliculus with varying degrees of stenosis or complete obstruction [2, 4]. These variations likely reflect differences in the mechanism and progression of drug-induced epithelial injury within the lacrimal drainage system. Therefore, a classification based solely on the presence or absence of obstruction may not sufficiently capture the diversity of canalicular pathology observed in patients receiving systemic chemotherapy.

Previous studies, including the classification proposed by Yabe and Suzuki, mainly focus on idiopathic canalicular obstruction and categorize the disease according to anatomic sites of obstruction [20]. While this approach has been useful in understanding primary canalicular disorders, drug-induced canalicular obstruction appears to have distinct clinical characteristics. In patients treated with S-1, epithelial toxicity caused by 5-fluorouracil metabolites is thought to induce subsequent fibrosis of the lacrimal drainage epithelium [21]. Because anti-cancer agents are excreted into tears, the entire canalicular lumen may be exposed to toxic metabolites, resulting in heterogeneous patterns of injury that do not always correspond to the typical focal obstruction patterns that occur in idiopathic disease.

A major challenge in the management of S-1/TDSD is that placement of a Jones tube requires lifelong maintenance and regular medical follow-up, which can impose a considerable burden on patients, particularly those living in remote areas. Therefore, achieving symptom relief without the need for Jones tube placement would provide substantial benefit. In the present study, incision of the horizontal canaliculus followed by exploration for a patent canaliculus for lacrimal tube insertion allowed avoidance of Jones tube placement in nearly one-half of the cases.

Another important issue in the management of S-1/TDSD is progressive canalicular sclerosis. Even when canalicular patency appears to be preserved, persistent epiphora may indicate the presence of canalicular stenosis or sclerosis. In such cases, preventive strategies may be required for high-risk patients with S-1/TDSD [6]. One potential approach is temporary occlusion of the lacrimal punctum to block lacrimal drainage, thereby reducing exposure of the lacrimal drainage system to tears containing S-1. Further studies are needed to evaluate the effectiveness of this strategy in high-risk patients.

From a short-term preventive perspective, most canalicular damage induced by cytotoxic anti-cancer drugs occurs within 3–4 months after initiation of therapy [5, 17]. Therefore, ophthalmologic examinations should be performed monthly during the first 4 months after the start of S-1 administration. If symptoms develop, lacrimal irrigation should be performed to evaluate ductal patency and to detect stenosis of both the upper and lower canaliculi.

Early surgical intervention should be considered before unilateral obstruction of the horizontal canaliculi develops. Several risk factors for S-1/TDSD are reported, including anorexia, oral mucositis, skin pigmentation, plasma 5-FU levels > 22.3 ng/mL, age > 70 years, creatinine clearance < 80 mL/min [5, 6], and high tegafur concentrations in tears [21]. For such high-risk patients, prophylactic tube placement or punctal plug insertion may reduce exposure of the lacrimal mucosa to S-1-containing tears and help prevent the progression of lacrimal drainage system damage.

### Study limitations

The limitations of this study include the retrospective, two-center design, the relatively small number of patients, the lack of histopathologic evaluations, and the inclusion of both sides in the same patient because of differences between the types of obstruction in a single patient with two affected sides. The small sample size and intermediate-term study may limit the generalizability of the findings.

Nevertheless, a 12-month follow-up period is considered clinically relevant because most surgical failures in lacrimal drainage procedures are reported to occur within the first postoperative year [22]. The favorable outcomes observed during this critical period suggest that the surgical strategies used in the present study provide stable short- to intermediate-term efficacy for S-1/TDSD.

Second, since the study was conducted at two institutions, selection bias could have occurred. The study was conducted after the onset of epiphora following S-1 administration; therefore, observations and accurate cumulative S-1 dose data before the onset of symptoms were not possible.

## Supplementary Information

Below is the link to the electronic supplementary material.

Supplementary file1 (TIF 2225 KB)

## Abbreviations

CO: Canalicular obstruction
EHC: Exploratory horizontal part canaliculotomy
